# Clinical observation of the efficacy of PD‐1/PD‐L1 inhibitors in the treatment of patients with advanced solid tumors

**DOI:** 10.1002/iid3.511

**Published:** 2021-08-18

**Authors:** Miao Wang, Hongchao Zhen, Xiaoyue Jiang, Yuting Lu, Yuhan Wei, Jiangtao Jin, Qin Li

**Affiliations:** ^1^ Department of Oncology, Beijing Friendship Hospital Capital Medical University Beijing China; ^2^ Department of Intervention Therapy Zezhou People's Hospital Jincheng China

**Keywords:** advanced solid tumor, clinical efficacy, efficacy prediction, immunotherapy, PD‐1/PD‐L1 inhibitor

## Abstract

**Introduction:**

Programmed death 1 (PD‐1)/programmed death‐ligand 1 (PD‐L1) inhibitors are proved to be promising and are applied for the treatment of a variety of solid tumors. This retrospective study evaluated the efficacy of PD‐1/PD‐L1 inhibitors in patients with advanced solid tumors and explore the effect of clinical characteristics on it.

**Materials and Methods:**

From October 2017 to April 2020, a total of 90 patients from Capital Medical University Affiliated Beijing Friendship Hospital were enrolled.

**Results:**

At a median follow‐up of 10.55 months, objective response was observed in 23 patients and the objective response rate was 25.6%. The median progression‐free survival (PFS) was 5.5 months (95% confidence interval [CI], 3.69–7.37). The 6m‐PFS was 45.8% and 12m‐PFS was 25.1%. The median overall survival (OS) was 16.9 months (95% CI, not reached [NR]‐NR). The 12m‐OS was 58.1% and 18m‐OS was 48.1%.

**Conclusion:**

The efficacy of PD‐1/PD‐L1 inhibitors in the treatment of advanced solid tumors was comparable to previous studies. ECOG performance status, smoking status, liver metastasis, neutrophil‐to‐lymphocyte ratio were independently correlated with PFS while liver metastasis and lactate dehydrogenase level were independently correlated with OS.

## Graphical abstract

we evaluate the efficacy of programmed death 1 (PD‐1)/programmed death‐ligand 1 (PD‐1/PD‐L1) inhibitors in patients with advanced solid tumors and explore the effect of clinical characteristics. ECOG status, smoking status, liver metastasis status, and NLR might predict the recurrence between PD‐1/PD‐L1 inhibitors treatment and liver metastasis, lactate dehydrogenase might predict the survival. This finding has great significance and value of the guidance for the application of immunotherapy.

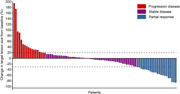

## 1 INTRODUCTION

Cancer is a major public health problem worldwide, with 18.1 million new cancer cases and 9.7 million cancer deaths annually.^1^ According to the estimation of the World Health Organization in 2015, cancer was the leading cause of death in 91 countries by the end of 21st century. The morbidity and mortality of cancer are increasing rapidly worldwide. Therefore, it has become an important problem to be solved to explore more active and effective treatment for cancer.

In recent years, a series of revolutionary changes have been made in tumor immunotherapy. Immune checkpoint inhibitors such as programmed death 1 (PD‐1)/programmed death‐ligand 1 (PD‐L1) inhibitors have been applied in a variety of solid tumors, like melanoma, lung cancer, renal cell carcinoma, head and neck squamous cell carcinoma, urothelial carcinoma, esophageal carcinoma, and other tumors, and regarded as the most promising treatment for malignant tumors.^2^, ^3^, ^4^, ^5^

Despite its significant and durable response in prospective clinical trials, only a small part of patients benefits from PD‐1/PD‐L1 inhibitors. It is essential to screen the beneficial population. Although biomarkers such as PD‐L1, tumor mutational burden (TMB), mismatch repair (MMR)/microsatellite instability (MSI) have been shown to be promising,^6^, ^7^, ^8^ there is still not a perfect biomarker to select patients to receive PD‐1/PD‐L1 inhibitors accurately. In addition, more real‐world analyses are necessary to guide the further application of these new therapies. Based on the hypothesis that the efficacy of PD‐1/PD‐L1 inhibitors on solid tumors in real life is comparable to that observed in clinical trials, this retrospective study evaluated the efficacy of PD‐1/PD‐L1 inhibitors in patients with advanced solid tumors and explore the effect of clinical characteristics.

## 2 MATERIAL AND METHODS

### 2.1 Patients

We conducted an observational retrospective analysis of patients with advanced solid tumors who received treatment with PD‐1/PD‐L1 inhibitor in Capital Medical University Affiliated Beijing Friendship Hospital between October 1, 2017 and April 1, 2020. Eligible criterial were as follows: (1) age ≥18 years; (2) histologically diagnosed malignant solid tumor; (3) blood routine, liver and kidney function is generally normal; (4) unresectable or multiple metastases; (5) at least one measurable or evaluable lesion according to Response Evaluation Criteria in Solid Tumors (RECIST), version 1.1^9^; (6) Eastern Cooperative Oncology Group (ECOG) performance status (PS) of 2 or less.

Patients were excluded if they had an autoimmune disease or were receiving any form of systemic immunotherapy or immunosuppressive therapy such as PD‐1/PD‐L1 inhibitors, interleukin, interferon, thymosin, or anti‐rejection drugs after organ transplantation.

This study was approved by the Ethics Committee for Clinical Investigation of Capital Medical University Affiliated Beijing Friendship Hospital and conducted according to the Declaration of Helsinki. All patients had signed informed consent for data collection and study purposes.

### 2.2 Treatment regimes

All the patients received PD‐1/PD‐L1 inhibitors monotherapy or combined with other treatment regimens every 3 weeks administered in accordance with guidelines until disease progression, unacceptable toxicity, or other termination criteria (pregnancy, patients' personal reasons, or intercurrent illness) were met. Other treatment regimens included chemotherapy and targeted therapy. Patients were evaluated every two or three cycles (6–9 weeks).

### 2.3 Data collection and efficacy assessment

The data collected included demographic data (gender, age, ECOG PS, history of smoking, family history of cancer), laboratory test of peripheral blood (lactate dehydrogenase [LDH], neutrophil‐to‐lymphocyte ratio [NLR] and platelet‐to‐lymphocyte ratio [PLR]), and treatment regime of PD‐1/PD‐L1 inhibitors (line, specific drug, and whether combined with others). The baseline characteristics of patients were derived from the medical records of patients at the beginning of immunotherapy. All data were identified before be released to the main investigator.

According to RECIST v1.1, the antitumor efficacy was evaluated by computed tomography (CT) or magnetic resonance imaging (MRI) every 2–3 treatment cycles, resulting in complete response (CR), partial response (PR), stable disease (SD), or progressed disease (PD). The objective was to describe the objective response rate (ORR), progression‐free survival (PFS), and overall survival (OS). Additionally, further analysis was conducted to explore the effect of clinical characteristics on the PFS and OS. PFS is defined as the time from the start of immunotherapy to disease progression/death or the date of their last disease assessment, whichever occurs first. OS was defined as the time from the beginning of PD‐1/PD‐L1 inhibitor treatment to death or to the final follow‐up time. Those who did not progress were reviewed on the date of their last disease assessment. DOR refers to the time from the first recorded response to tumor progression or death or the last evaluation for patients with target response.

### 2.4 Statistical analysis

The Kaplan–Meier approach was used to estimate PFS, OS, and DOR and 95% confidence interval (CI). Patients who lost follow‐up were reviewed on the date of last follow‐up time. PFS of subgroups were compared with the log‐rank test to determine the relationship between patients' clinical characteristics (age, gender, smoking status, ECOG PS, systemic metastasis, etc.) and recurrence outcomes. Cox regression was performed to estimate the hazard ratios (HRs) and 95% CI. *p* < .05 was considered statistically significant. The follow‐up deadline is January 18, 2021. All statistical analyses were performed using SPSS 25.0 software.

## 3 RESULTS

### 3.1 Patient characteristics

A total of 90 patients were enrolled in the study, including 58 males (64.4%) and 32 females (35.6%). The mean age was 61.97 ± 8.44 years old, ranging from 31 to 77 years old. The baseline characteristics of the patients are shown in Table 1. Primary tumor sites were distributed throughout the body, involving a total of 17 tumor types, including 22 cases of NSCLC (18 cases of adenocarcinoma, 4 cases of squamous cell carcinoma), 19 cases of esophageal cancer, 12 cases of gastric cancer, 7 cases of colorectal cancer, 6 cases of small‐cell lung cancer, and 24 cases of other tumor types. There were 15 patients with stage III and 75 patients with stage IV. Most patients had an ECOG PS of 1 (*N* = 62, 68.9%), 17 patients were 0 and 11 patients were 2. 33.3% (30/90) of the patients received resection of the primary lesion, 8 patients (8.9%) had brain metastasis, and 23 patients (25.6%) had liver metastasis.

**Table 1:** Baseline characteristics and univariate analysis of PFS and OS for all patients

Characteristics	*N*	PFS (95% CI, mo)	*p*	OS (95% CI, mo)	*p*
Gender			.154		.614
Male	58	5.73 (3.16–8.30)		NR (NR–NR)	
Female	32	4.27 (1.61–6.94)		12.57 (7.77–17.37)	
Age			.335		.001
<65	51	5.93 (3.69–8.17)		NR (NR–NR)	
≥65	39	4.53 (1.61–7.46)		10.67 (6.78–14.56)	
ECOG PS			.013		.026
0	17	7.00 (NR–NR)		NR (NR–NR)	
1	62	4.97 (3.32–6.62)		12.57 (3.59–21.55)	
2	11	2.90 (0.51–5.29)		8.97 (5.47–12.47)	
Smoking history			.018		.040
No	44	4.00 (2.71–5.29)		12.4 (7.32–17.48)	
Yes	46	7.37 (6.13–8.61)		NR (NR–NR)	
Drinking history			.513		.518
No	59	5.10 (3.29–6.91)		12.57 (NR–NR)	
Yes	31	6.73 (3.12–10.34)		NR (NR–NR)	
Family history of tumor			.092		.511
No	70	5.10 (3.74–6.47)		16.40 (11.02–21.78)	
Yes	20	13.20 (0.15–26.25)		NR (NR–NR)	
Tumor types			<.001		.008
NSCLC	22	5.53 (0.96–10.10)		NR (NR–NR)	
EC	19	4.97 (1.07–8.87)		9.43 (1.53–17.33)	
GC	12	5.73 (3.25–8.21)		NR (NR–NR)	
CRC	7	1.63 (0.99–2.27)		5.63 (5.22–6.04)	
SCLC	6	4.53 (0.47–8.59)		8.67 (2.24–15.10)	
Others	24	6.97 (3.07–10.87)		NR (NR–NR)	
Stage			.077		.197
III	15	NR (NR–NR)		NR (NR–NR)	
IV	75	4.97 (3.62–6.32)		12.57 (NR–NR)	
Brain metastasis			.750		.908
No	82	5.53 (4.07–6.99)		16.90 (NR–NR)	
Yes	8	6.73 (3.26–10.20)		12.57 (8.38–16.77)	
Liver metastasis			<.001		.002
No	67	6.93 (5.75–8.11)		NR (NR–NR)	
Yes	23	3.07 (2.10–4.04)		8.13 (5.79–10.47)	
Lung metastasis			.087		.038
No	64	5.93 (3.34–8.52)		NR (NR–NR)	
Yes	26	5.10 (1.45–8.75)		8.83 (6.37–11.29)	
Bone metastasis			.278		.092
No	65	6.73 (5.19–8.28)		NR (NR–NR)	
Yes	25	4.53 (4.15–14.91)		11.23 (4.70–17.76)	
NLR			.009		.045
<4	52	6.93 (5.58–8.28)		NR (NR–NR)	
≥4	38	4.13 (3.02–5.24)		11.23 (7.06–15.40)	
PLR			.235		.335
<224	51	5.93 (2.98–8.88)		NR (NR–NR)	
≥224	39	4.97 (3.36–6.58)		16.40 (5.90–26.90)	
LDH			.069		.001
<ULN	70	6.73 (5.36–8.10)		NR (NR–NR)	
≥ULN	20	4.27 (2.61–5.93)		8.07 (6.75–9.39)	
PD‐1 types			.635		.242
Sintilimab	41	6.97 (3.56–10.38)		NR (NR–NR)	
Camrelizumab	19	6.73 (0.39–13.07)		10.67 (7.53–13.81)	
Others	30	5.53 (3.93–7.13)		16.40 (6.30–26.50)	
Line			.771		.390
1	28	5.73 (3.28–8.18)		NR (NR–NR)	
2	34	5.93 (3.92–7.94)		16.90 (6.42–27.38)	
3+	28	3.30 (2.60–4.00)		12.57 (NR–NR)	
MONO or COM			.658		.327
MONO	20	5.93 (1.91–9.95)		NR (NR–NR)	
COM	70	5.10 (3.00–7.20)		12.57 (6.08–19.06)	

Abbreviations: COM, combination therapy; ECOG PS, Eastern Cooperative Oncology Group performance status; LDH, lactate dehydrogenase; MONO, monotherapy; NLR, neutrophil‐to‐lymphocyte ratio; NR, not reached; OS, overall survival; PFS, progression‐free survival; PLR, platelet‐to‐lymphocyte ratio; ULN, upper limit of normal.

Forty‐one patients (45.6%) received sintilimab at a dose of 200 mg every 3 weeks; 19 patients (21.1%) received camrelizumab at a dose of 200 mg every 3 weeks. The rest of the 30 cases of patients accepted other PD‐1/PD‐L1 inhibitors such as nivolumab, atezolizumab, etc. All drugs are used in accordance with the instructions. Most patients received PD‐1/PD‐L1 inhibitors combined with other treatment strategies (*N* = 70, 77.8%), and the rest received monotherapy. The median number of treatment lines for all patients was two (range: 1–7), among which 28 patients received first‐line treatment, 34 patients received second‐line treatment, and the remaining 28 patients received third‐line or more than third‐line treatment. None of the patients received reduced dose treatment.

### 3.2 Efficacy and safety of total patients

**Figure 1:**
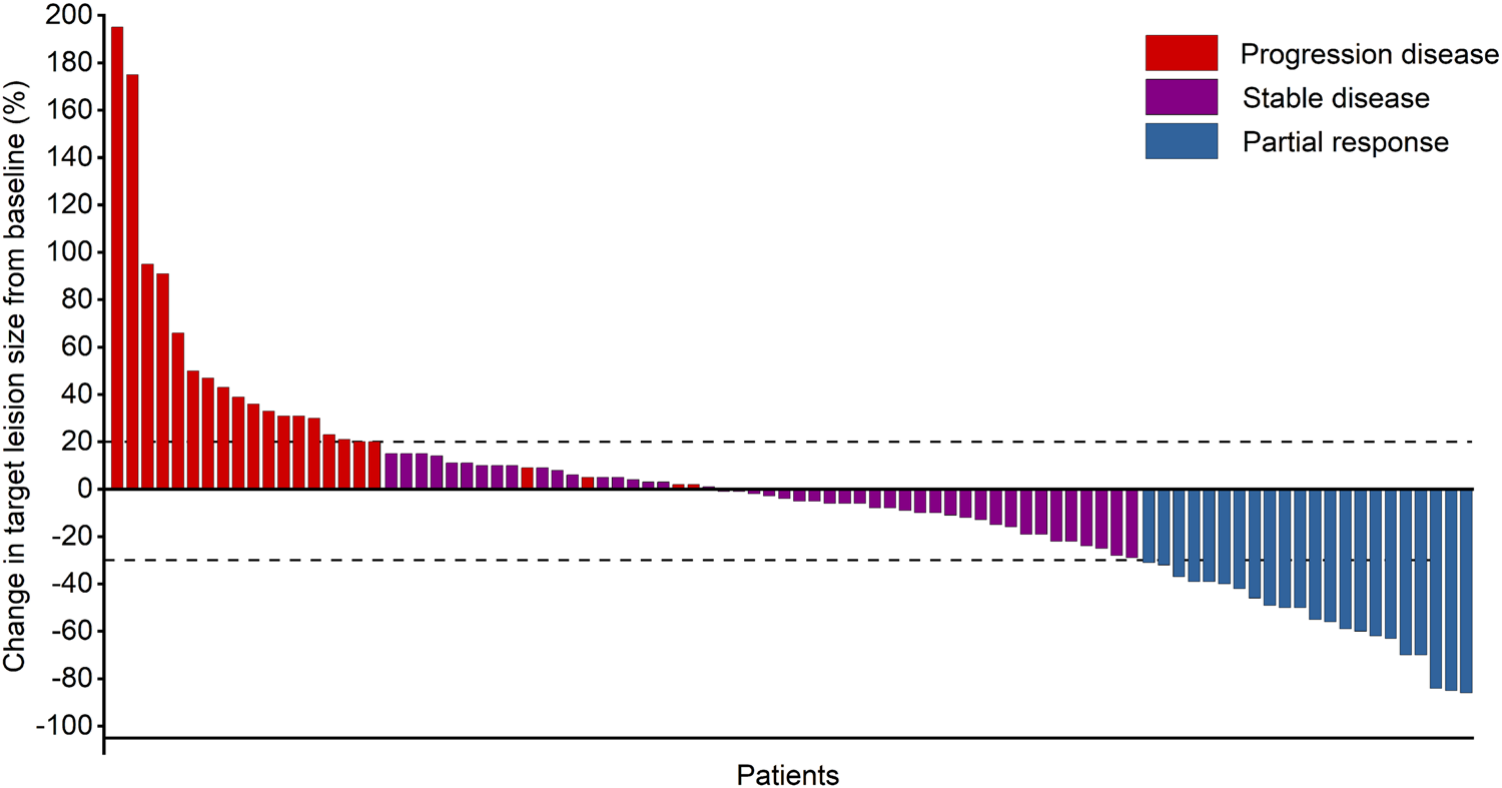
Waterfall plots show the maximum percentage change in target lesion size during active treatment with PD‐1/PD‐L1 inhibitors. PD‐1/PD‐L1, programmed death 1 (PD‐1)/programmed death‐ligand 1

**Figure 2:**
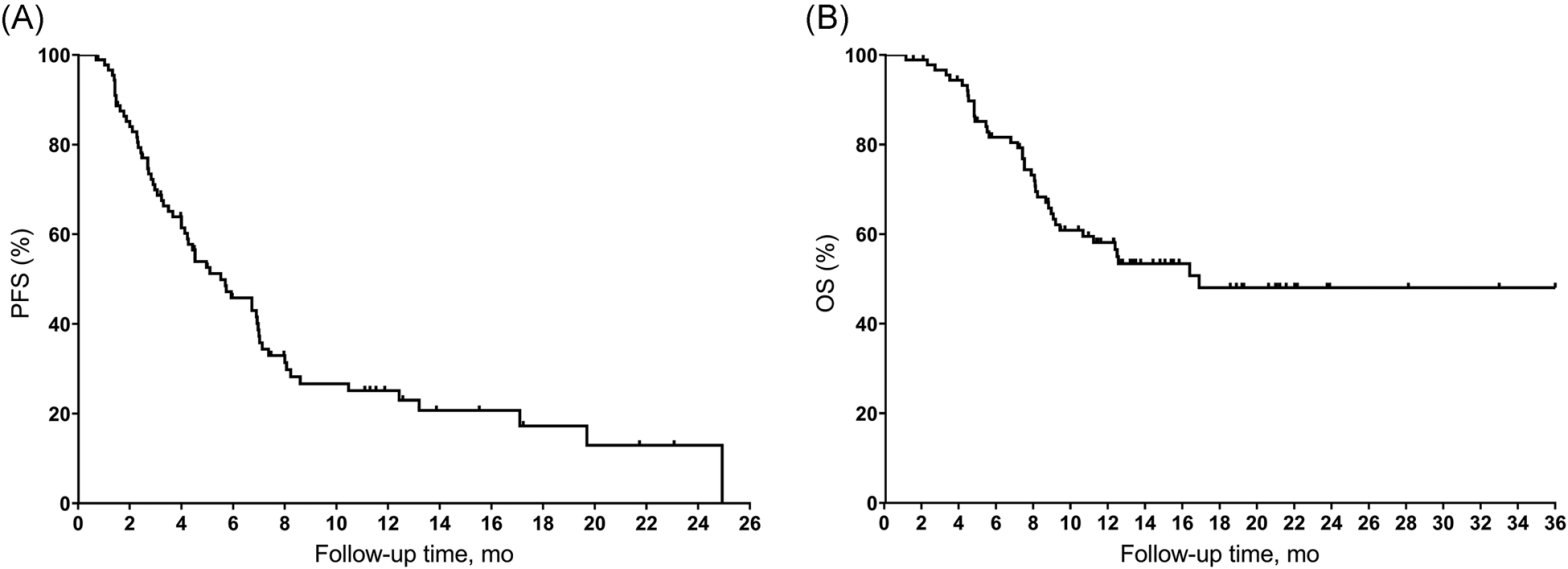
Kaplan–Meier plots of median (A) progression‐free survival (PFS) and (B) overall survival (OS) in all populations

The median follow‐up time was 10.6 months (range: 1.2–36.0 months; interquartile range [IQR]: 6.54–15.43). Overall, CR was not observed, and 23 patients achieved PR (Figure 1). The ORR and DCR were 25.6% and 75.6%, respectively. Among the 23 responders, the median DOR was 3.47 months (range: 0.07–14.23 months). The median PFS was 5.5 months (95% CI, 3.69–7.37) and median OS was 16.9 months (95% CI, not reached [NR]–NR). The 6m‐PFS was 45.8% and 12m‐PFS was 25.1%. The 6m‐OS was 81.6%, 12m‐OS was 58.1%, 18m‐OS was 48.1%. The Kaplan–Meier curves of PFS and OS were shown in Figure 2.

Immune‐related adverse events (irAEs) were developed in patients who received monotherapy and combination. In monotherapy group, two (10.00%) patients developed grade 1–2 rash, and no patients developed grade ≥3 irAEs. In combination therapy group, 7 (10.00%) patients developed pneumonia in grades 1–2, one patient developed grade ≥3 rash. Many other adverse events were also developed in monotherapy and combination therapy which were shown in Table 2. And six patients discontinued therapy due to any grade 3–5 adverse events.

**Table 2:** Treatment‐related adverse events

	Monotherapy (*n* = 20)		Combination therapy (*n* = 70)	
Adverse events	Grade 1–2	Grade ≥3	Grade 1–2	Grade ≥3
Immune‐related adverse events				
Rash	2 (10.00%)	0 (0.00%)	3 (4.29%)	1 (1.43%)
Diarrhea	2 (10.00%)	0 (0.00%)	0 (0.00%)	0 (0.00%)
Hypothyroidism	1 (5.00%)	0 (0.00%)	3 (4.29%)	0 (0.00%)
Hyperthyroidism	1 (5.00%)	0 (0.00%)	3 (4.29%)	0 (0.00%)
Heart‐related symptoms	1 (5.00%)	0 (0.00%)	3 (4.29%)	0 (0.00%)
Pneumonia	1 (5.00%)	0 (0.00%)	7 (10.00%)	0 (0.00%)
Hepatitis	1 (5.00%)	0 (0.00%)	0 (0.00%)	0 (0.00%)
Herpes zoster	1 (5.00%)	0 (0.00%)	1 (1.43%)	0 (0.00%)
Reactive cutaneous capillary endothelial proliferation	0 (0.00%)	0 (0.00%)	0 (0.00%)	1 (1.43%)
Arthralgia	0 (0.00%)	0 (0.00%)	1 (1.43%)	0 (0.00%)
Other adverse events				
Anemia	2 (10.00%)	2 (10.00%)	18 (25.71%)	3 (4.29%)
Hypocalcemia	4 (20.00%)	0 (0.00%)	18 (25.71%)	0 (0.00%)
Fatigue	3 (15.00%)	0 (0.00%)	7 (10.00%)	0 (0.00%)
Decreased white‐cell count	3 (15.00%)	0 (0.00%)	20 (28.57%)	3 (4.29%)
Hypokalemia	3 (15.00%)	0 (0.00%)	16 (22.86%)	1 (1.43%)
Decreased appetite	2 (10.00%)	0 (0.00%)	6 (8.57%)	0 (0.00%)
Nausea	2 (10.00%)	0 (0.00%)	5 (7.14%)	0 (0.00%)
Decreased platelet count	2 (10.00%)	0 (0.00%)	7 (10.00%)	2 (2.86%)
Stomatitis	1 (5.00%)	0 (0.00%)	0 (0.00%)	0 (0.00%)
Constipation	1 (5.00%)	0 (0.00%)	3 (4.29%)	0 (0.00%)
Alopecia	1 (5.00%)	0 (0.00%)	0 (0.00%)	0 (0.00%)
Decreased neutrophil count	0 (0.00%)	1 (5.00%)	7 (10.00%)	10 (14.29%)
Increased aspartate aminotransferase	5 (25.00%)	0 (0.00%)	7 (10.00%)	1 (1.43%)
Increased alanine aminotransferase	2 (10.00%)	0 (0.00%)	10 (14.29%)	0 (0.00%)
Increased blood creatinine	1 (5.00%)	0 (0.00%)	5 (7.14%)	1 (1.43%)
Pruritus	0 (0.00%)	0 (0.00%)	2 (2.86%)	0 (0.00%)
Vomiting	0 (0.00%)	0 (0.00%)	10 (14.29%)	0 (0.00%)
Peripheral edema	0 (0.00%)	0 (0.00%)	1 (1.43%)	0 (0.00%)

### 3.3 Subgroup analysis for effects exploration

**Figure 3:**
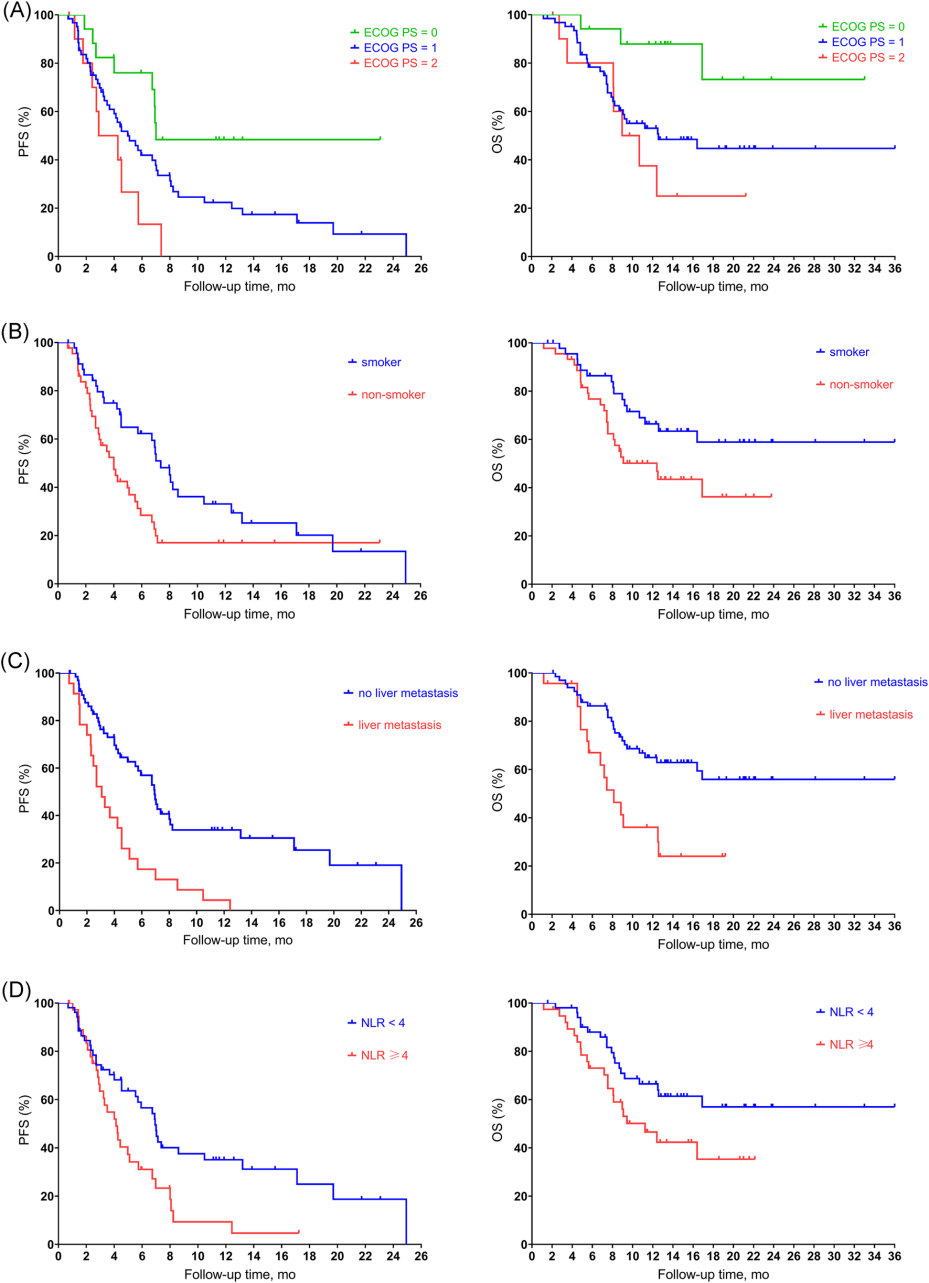
Kaplan–Meier estimates of PFS and OS by (A) ECOG, (B) liver metastasis, (C) smoking status, (D) NLR value. ECOG, Eastern Cooperative Oncology Group; NLR, neutrophil‐to‐lymphocyte ratio; OS, overall survival; PFS, progression‐free survival

Univariate analysis of patients' baseline showed that patients with ECOG PS = 0 had a median PFS of 7.0 months (95% CI: NR–NR), while patients with ECOG PS = 1 had a PFS of 5.0 months (95% CI: 3.32–6.61), and patients with ECOG PS = 2 had a PFS of 2.9 months (95% CI: 0.51–5.29). The differences among different ECOG PS were statistically significant (*p* = .013). As for survival data, the OS of the three groups were NR (95% CI: NR–NR), 12.6 months (95% CI: 3.59–21.55), 9.0 months (95% CI: 5.47–12.47) and the differences were statistically significant (*p* = .026; Figure 3). In addition, compared with patients who never smoked (mPFS: 4.0 months, 95% CI: 2.71–5.29; mOS: 12.4 months, 95% CI: 7.32–17.48), patients who smoked had significantly longer PFS (7.4 months, 95% CI: 6.13–8.61; *p* = .018) and OS (NR, 95% CI: NR–NR; *p* = .040). Liver metastasis was negatively correlated with PFS (liver metastasis: 3.1 months, 95% CI: 2.10–4.04; no liver metastasis: 6.9 months, 95% CI: 5.75–8.11; *p* < .001) and OS (liver metastasis: 8.1 months, 95% CI: 5.79–10.47; no liver metastasis: NR, 95% CI: NR–NR; *p* = .002). Patients with NLR higher than 4 had significantly shorter mPFS than those with NLR lower than 4 (NLR < 4: 6.9 months, 95% CI: 5.58–8.28; NLR ≥ 4: 4.1 months, 95% CI: 3.02–5.24) and the difference was statistically significant (*p *= .009). The survival data was also significant (NLR < 4: NR, 95% CI: NR‐NR; NLR ≥ 4: 11.2 months, 95% CI: 7.06–15.40; *p *= .045).

There were no statistically significant different between PFS, OS, and other factors such as gender, age, stage, monotherapy/combination therapy, history of drinking, family history of tumor, brain metastasis, lung metastasis, bone metastasis, PLR, and LDH, etc. shown in Table 1.

### 3.4 Multivariate analyses

Multivariate Cox regression showed that ECOG PS score, smoking state, liver metastasis, NLR were independently correlated with PFS, and liver metastasis, LDH level were independently correlated with OS, as shown in Table 3.

**Table 3:** Multivariate analysis of explored covariates with survival outcomes

Characteristics	PFS		OS	
	HR (95% CI)	*P*	HR (95% CI)	*P*
Age	1.01 (0.972–1.039)	.776	1.04 (0.999–1.089)	.053
≥65 vs. <65				
ECOG PS	2.32 (1.374‐3.928)	.002	1.68 (0.900–3.141)	.103
2 vs. 1 vs. 0				
Smoking history	0.36 (0.203–0.623)	<.001	0.54 (0.284–1.035)	.063
Yes vs. no				
Liver metastasis	2.89 (1.688–4.943)	<.001	2.59 (1.323–5.079)	.006
Yes vs. no				
NLR	2.00 (1.172–3.414)	.011	1.74 0.910–3.310)	.094
≥4 vs. <4				
LDH	1.08 (0.595–1.953)	.805	2.15(1.030–4.477)	.041
≥ULN vs. ULN				

Abbreviations: ECOG PS, Eastern Cooperative Oncology Group performance status; LDH, lactate dehydrogenase; NLR, neutrophil‐to‐lymphocyte ratio; OS, overall survival; PFS, progression‐free survival; ULN, upper limit of normal.

### 3.5 Efficacy analysis according to tumor types

Tumor types of more than 10 patients were analyzed including 22 cases of NSCLC, 19 cases of esophagus cancer, and 12 cases of gastric cancer. For NSCLC, 5 patients received first‐line treatment, and 17 patients received second or more line treatment. Six (27.3%) of 22 participants in the NSCLC group had an objective response. No patient had a complete response, 6 patients were PR, 14 patients were SD, 2 patients were PD. ORR was 27.3%, and DCR was 90.9%. The median PFS was 5.5 months (95% CI: 0.96–10.10), 6m‐PFS was 48.9% and 12m‐PFS was 36.7%. The median OS was not reached, 6m‐OS was 90.9%, 12m‐OS was 65.9%, and 18m‐OS was 54.9%. Nineteen patients were diagnosed with esophageal carcinoma: 8 patients received first‐line treatment and 11 patients received second or more line treatment. Seven patients achieved PR, 7 patients with SD, and 5 patients with PD. The ORR was 36.8%, and DCR was 73.6%. The median PFS was 5.0 months (95% CI: 1.07–8.87), 6m‐PFS was 48.1% and 12m‐PFS was 16.0%. The median OS was 9.4 months (95% CI: 1.53–17.33), 6m‐OS was 73.3%, 12m‐OS was 48.9%, and 18m‐OS was 29.3%. For GC, 7 patients received first‐line treatment and 5 patients received second or more line treatment. No CR, 3 patients with PR, 7 patients with SD, 2 patients with PD, ORR was 25.0%, and DCR was 83.3%. The median PFS was 5.7 months (95% CI: 3.25–8.21), 6m‐PFS was 32.1% and 12m‐PFS was 21.4%. The median OS was not reached, 6m‐OS was 90.0%, 12m‐OS was 70.0%, and 18m‐OS was 70.0% (Table 4).

**Table 4:** Subgroup analysis of efficacy according to tumor types

	Total	NSCLC	EC	GC
Total	90	22	19	12
Best response				
CR	0	0	0	0
PR	23	6	7	3
SD	45	14	7	7
PD	22	2	5	2
ORR (%)	25.6	27.3	36.8	25
DCR (%)	75.6	90.9	73.6	83.3
mPFS (mo, 95% CI)	5.53 (3.69–7.37)	5.53 (0.96–10.10)	4.97 (1.07–8.87)	5.73 (3.25–8.21)
6m‐PFS (%)	45.8	48.9	48.1	32.1
12m‐PFS (%)	25.1	36.7	16	21.4
mOS (mo, 95% CI)	16.9 (NR–NR)	NR	9.43 (1.53–17.33)	NR
6m‐OS (%)	81.6	90.9	73.3	90.0
12m‐OS (%)	58.1	65.9	48.9	70.0
18m‐OS (%)	48.1	54.9	29.3	70.0

Abbreviations: CR, complete response; DCR, disease control rate; EC, esophageal cancer; GC, gastric cancer; mPFS, median progression‐free survival; mOS, median overall survival; NR, not reached; NSCLC, non‐small‐cell lung cancer; ORR: overall response rate; PD: progressive disease; PR, partial response; SD, stable disease.

## 4 DISCUSSION

PD‐1/PD‐L1 inhibitor has become one of the most important treatment for cancer, completely changing the strategy of tumor treatment. Several clinical trials have evaluated the efficacy and safety of PD‐1/PD‐L1 inhibitors in patients with solid tumors, showing durable efficacy and controllable safety.^10^ However, there are strict inclusion criteria for clinical trials. It is inevitable for the wider use of new regimes out of the eligibility criteria for clinical trials after the approval of authorities, especially when there are not attractive choice and standard treatment has considerable toxicity and poor efficacy. Therefore, real‐world evidence is necessary to prove the efficacy of a newly approved drug.

Our single‐center retrospective study assessed the efficacy of PD‐1/PD‐L1 inhibitors monotherapy or in combination with other treatment strategies (chemotherapy or antiangiogenesis inhibitor) for 90 patients with advanced solid tumors. The ORR was 25.6% among all patients, with all 23 patients having a partial response. The median PFS was 5.5 months and OS was 16.9 months. The 6m‐PFS was 45.8% and 12m‐PFS was 25.1%. The 12m‐OS and 18m‐OS were 58.1% and 48.1%, respectively. Our results are generally consistent with the results of previously published prospective studies, which showed the ORR of PD‐1/PD‐L1 inhibitors checkpoint inhibitors ranged from 20% to 30% for solid tumors.^11^ We collected the irAEs and other adverse events of 90 patients and classified different occurrence frequencies in monotherapy and combination therapy. And more study should be conducted to explore the management of these adverse events.

We respectively analyzed the major tumor types involved in the study. For NSCLC, previous published clinical trials showed that ORR ranged from 27.2% to 62.6%, PFS ranged from 5.1 to 9.0 months, and OS ranged from 16.4 to 26.3 months.^12^, ^13^, ^14^ Our study showed that ORR was 27.3%, PFS was 5.5 months (95% CI: 0.96–10.10) and the median OS was not reached. It was shown that ORR ranged from 9.9% to 46%, PFS ranged from 1.7 to 6.3 months, and OS ranged from 7.1 to 12.4 months for esophageal cancer in previous study^4^, ^15^, ^16^ and they were 36.8%, 5.0 months (95% CI: 1.07–8.87) and 9.4 months (95% CI: 1.53–17.33), respectively in our study. For GC patients, ORR, mPFS, and mOS were 25.0%, 5.7 months, and NR in the study, compared with 2.2%–57.5% for ORR, 1.4–10.45 months for PFS, and 4.6–9.1 months for OS in published studies.^17^, ^18^, ^19^

Furthermore, we explored the effect of clinical characteristics (gender, smoking status, liver metastasis status, inflammatory response, etc.) on PFS and OS. One of the most interesting finding in the study was that patients who smoked had significantly longer PFS and OS compared with patients who never smoked, which was consistent with previous study.^20^ It is demonstrated that smoking can cause many genetic mutations and higher PD‐L1 expression level,^21^ which may account for the difference of efficacy between different smoking status.

We also explored the relationship between inflammation and the PFS of PD‐1/PD‐L1 inhibitors based on the phenomenon that inflammation is known as the main driver of tumor development and has prognostic value in several kinds of malignant tumors.^22^ Among all the simple and easily detected biomarkers, NLR and PLR reflect the level of systemic inflammatory response, which is significantly correlated with the occurrence, development, and prognosis of various malignant tumors such as lung cancer, gastric cancer, colorectal cancer, breast cancer, and thyroid cancer, and can predict the risk of tumor recurrence and metastasis. In addition, several studies showed that there was also a significant correlation between NLR, PLR and the efficacy PD‐1/PD‐L1 inhibitors.^23^ Our retrospective study showed that NLR was associated with the PFS and OS of PD‐1/PD‐L1 inhibitors, while PLR was not. Therefore, NLR may be taken into consideration for the use of PD‐1/PD‐L1 inhibitors.

There were no statistically significant different between PFS, OS and other factors such as gender, age, stage, monotherapy/combination therapy, history of drinking, family history of tumor, brain metastasis, lung metastasis, bone metastasis, PLR, and LDH, etc., which was consistent with previous studies.^24^, ^25^, ^26^, ^27^, ^28^

Although the discovery was interesting, the explanation for the results should be carefully, given the fact that there were several inevitable limitations. The main limitation was that it may introduce unexpected deviation because it was a retrospective design such as the bias in the doctor's decision. Secondly, the power of the study to a certain extent is limited to the limited sample size and follow‐up time, which may lead to some errors in our results. Therefore, additional investigations will be required to verify the results. In addition, the heterogeneity of the study was relatively large, which is acceptable considering that we are trying to explore the efficacy of PD‐1/PD‐L1 inhibitors and its effect on solid tumors from an overall perspective.

In summary, this study showed that PD‐1/PD‐L1 inhibitors showed comparable efficacy in the real world compared with studies for advanced solid tumors. ECOG status, smoking status, liver metastasis status, and NLR might predict the recurrence of between PD‐1/PD‐L1 inhibitors treatment and liver metastasis, LDH might predict the survival. This finding has great significance and value of the guidance for the application of immunotherapy.

## CONFLICT OF INTERESTS

The authors declare that there are no conflict of interests.

## AUTHOR CONTRIBUTIONS

Qin Li contributed to the design of the study and was responsible for the integrity of the data and accuracy of the data analysis. Miao Wang, Hongchao Zhen, and Xiaoyue Jiang collected and extracted the clinical data. Miao Wang, Yuting Lu, and Yuhan Wei were responsible for the statistical analysis. Miao Wang, Hongchao Zhen, and Jiangtao Jin wrote the manuscript. All authors contributed to the review and approved the paper. All authors agreed to be accountable for the content of this paper.

## Data Availability

All data generated or analyzed during this study are included in this published article (and its supplementary materials).
